# Rush Medical College

**Published:** 1855-12

**Authors:** 


*Rush Medical College.*—In the introductory lecture of Prof. Brainard, contained in this number of the Journal, the reader will find an interesting history of this institution from its foundation to the present time. We will only add that the number in attendance, thus far this session is one hundred and forty; a very gratifying increase over several previous years.

